# MiR-21 Is a Novel Diagnostic and Prognostic Circulating Biomarker in Pleural Mesothelioma

**DOI:** 10.3390/diagnostics16081142

**Published:** 2026-04-11

**Authors:** Berta Mosleh, Yawen Dong, Elisabeth Lang, Thomas Klikovits, Katharina Sinn, Steven Kao, Marko Jakopovic, Clemens Aigner, Balazs Hegedüs, Natalie Baldes, Servet Bölükbas, Balazs Dome, Mir Alireza Hoda, Viktoria Laszlo, Michael Grusch, Karin Schelch

**Affiliations:** 1 Department of Thoracic Surgery, Medical University of Vienna, 1090 Vienna, Austria; berta.mosleh@meduniwien.ac.at (B.M.); yawen-dong@hotmail.com (Y.D.); thomas.klikovits@meduniwien.ac.at (T.K.); katharina.sinn@meduniwien.ac.at (K.S.); clemens.aigner@meduniwien.ac.at (C.A.); balazs.dome@meduniwien.ac.at (B.D.); mir.hoda@meduniwien.ac.at (M.A.H.); viktoria.laszlo@meduniwien.ac.at (V.L.); 2 Center for Cancer Research, Medical University of Vienna, 1090 Vienna, Austriamichael.grusch@meduniwien.ac.at (M.G.); 3 Department of Thoracic Surgery, Karl Landsteiner Institute for Clinical and Translational Thoracic Surgery Research, Clinic Floridsdorf, 1210 Vienna, Austria; 4 Sydney Medical School, University of Sydney, Camperdown, NSW 2050, Australia; steven.kao@sydney.edu.au; 5 Department for Respiratory Diseases Jordanovac, University Hospital Centre Zagreb, School of Medicine, University of Zagreb, 10000 Zagreb, Croatia; mjakopov@kbc-zagreb.hr; 6 Department of Thoracic Surgery, University Medicine Essen, University Duisburg-Essen, 45239 Essen, Germany; balazs.hegedues@rlk.uk-essen.de (B.H.); natalie.baldes@rlk.uk-essen.de (N.B.); servet.boeluekbas@rlk.uk-essen.de (S.B.); 7 National Koranyi Institute of Pulmonology, 1121 Budapest, Hungary; 8 Department of Thoracic Surgery, National Institute of Oncology, Semmelweis University, 1122 Budapest, Hungary

**Keywords:** pleural mesothelioma, microRNA-21, circulating biomarker, SMRP

## Abstract

**Background/Objective:** The identification of novel non-invasive diagnostic and prognostic biomarkers is urgently needed in pleural mesothelioma (PM). While soluble mesothelin-related peptides (SMRP) are the most established circulating biomarker, their prognostic value is limited. A wide range of microRNAs (miRs) play diverse roles in regulating gene expression in PM. MiR-21 has been shown to be upregulated in mesothelioma tissue; nevertheless, the diagnostic and prognostic utility of miR-21 in the circulation and its association with survival in PM have not been extensively investigated to date. The objective of the current study was to evaluate miR-21 as a potential blood-based diagnostic and prognostic biomarker in PM. **Methods:** Plasma samples from PM patients (*n* = 94) were collected at the time of diagnosis, prior to treatment. Sex- and age-matched healthy individuals (*n* = 30) served as controls. MiR-21 levels were measured using quantitative RT-PCR and normalized to miR-16, and potential correlations with clinicopathological data were analyzed. Serum SMRP levels were measured in matched patients *(n* = 84), and a direct comparative analysis of miR-21 and SMRP was conducted. In situ hybridization (ISH) was used to confirm the presence of miR-21 in tumor cells. **Results:** Plasma miR-21 levels were significantly elevated in PM patients compared to healthy controls (*p* < 0.001), demonstrating good diagnostic performance (AUC 0.81). The localization of miR-21 in PM cells was confirmed by ISH. High miR-21 levels were associated with significantly shorter median overall survival (12.4 vs. 24.3 months, *p* < 0.001). Elevated SMRP levels were also associated with reduced survival (12.4 vs. 19.5 months, *p* = 0.032); however, SMRP did not retain independent prognostic significance in multivariable analysis. In contrast, high-circulating miR-21 was confirmed as an independent predictor for poor survival (HR 3.12, *p* < 0.001). **Conclusions:** Our findings highlight that circulating miR-21 is a potential non-invasive biomarker with both diagnostic and independent prognostic value in pleural mesothelioma and outperforms SMRP in multivariable survival analysis. Further research is warranted to validate its role in the biology of this disease and to assess its correlation with outcome and treatment responses.

## 1. Introduction

Pleural mesothelioma (PM) is a highly aggressive asbestos-related malignancy. The prognosis is poor, with a median overall survival of 8–20 months, depending on the histology, stage, and treatment [1]. The disease poses significant diagnostic and therapeutic challenges. Early detection is difficult, and diagnoses are frequently made in advanced stages. Novel treatment options, such as checkpoint inhibitors, while offering some improvements, often have limited benefits in terms of survival [2,3,4]. To date, no established formal screening guidelines exist in high-risk populations, except for carriers of germline BRCA-1-associated protein (BAP1) mutations. For definitive diagnosis, surgical tissue biopsy remains the primary method [5,6]. While some blood-based biomarkers, such as soluble mesothelin-related peptide (SMRP) and calretinin, have been demonstrated to be potentially useful as diagnostic blood-based biomarkers in mesothelioma, non-invasive diagnosis remains an ongoing challenge [7,8,9,10]. Among circulating biomarkers, SMRP is the most extensively investigated and remains the only Food and Drug Administration (FDA)-approved blood-based biomarker. SMRP is primarily used as an adjunctive diagnostic marker and for longitudinal disease monitoring, including assessment of treatment response and detection of recurrence, with its clinical utility being most pronounced in patients with epithelioid or biphasic histological subtypes. While SMRP is the most established circulating biomarker, its prognostic value is limited. Overall, circulating biomarkers have been characterized by poor sensitivity and limited diagnostic potential, particularly in the early disease stages and in sarcomatoid histology [11,12,13,14,15]. In the era of precision oncology, novel circulating biomarkers are urgently needed to facilitate an early and less invasive diagnosis, particularly in high-risk individuals with a history of asbestos exposure. The search for non-invasive circulating biomarkers to aid in diagnosis and prognostication in pleural mesothelioma continues.

MicroRNAs (miRs) are small, non-coding RNA molecules with a crucial role in gene expression regulation. The dysregulation of miRs has been reported in various malignancies [16,17,18,19,20]. In pleural mesothelioma, miRs have been shown to exhibit an altered expression pattern, with both upregulation and downregulation observed [21,22,23,24,25]. MiR-16, miR-193a, and miR-215 were shown to have tumor suppressor activity, while miR-24-3p, miR-182-5p, and miR-183-5p were found to be oncogenic [26,27].

MiR-21 is a well-characterized oncogenic microRNA involved in tumor progression, immune modulation, and treatment resistance across multiple malignancies. Its dysregulation has been associated with advanced disease stages and adverse clinical outcomes [28,29,30]. In the immune system, both pro- and anti-inflammatory effects have been observed, promoting inflammatory mediators, modulating T-cell responses, and influencing macrophage function [31,32]. High expression of miR-21 was also found to be correlated with the pathogenesis of autoimmune disorders and allograft dysfunction after organ transplantation [33,34].

To date, the correlation between circulating miR-21, survival, and treatment has not been investigated. Also, its relevance as a potential circulating biomarker relative to SMRP has not been evaluated so far.

In the current study, we further investigate the diagnostic and prognostic potential of circulating miR-21 and its association with survival outcome, and compare its clinical value with that of SMRP to evaluate its suitability as a blood-based biomarker in pleural mesothelioma. In addition, in situ hybridization (ISH) was performed to confirm that the detected miR-21 signal originates from tumor cells.

## 2. Materials and Methods

### 2.1. Patients and Patient Material

Institutional biobanks were reviewed for patients with histologically confirmed pleural mesothelioma with available samples, where plasma and serum of 94 patients (male 79%, median age 64 years, IQR: 56–64) were retrospectively analyzed for circulating microRNAs and Soluble Mesothelin-Related Peptides (SMRP), respectively. Seventy samples were obtained at the Department of Thoracic Surgery at the Medical University of Vienna, Austria. Nineteen samples were collected at the University of Zagreb, School of Medicine, Department for Respiratory Diseases, Jordanovac, University Hospital Center Zagreb, Croatia. Five samples were acquired at the Sydney Medical School, The University of Sydney, Sydney, NSW, Australia. The control group consisted of 30 sex- and age-matched healthy individuals (male 77%, median age 64 years, IQR: 59–69). Healthy controls were obtained from institutional biobank collections and consisted of individuals without known malignant disease, chronic inflammatory conditions, or active pulmonary disease at the time of blood sampling. Available clinical data confirmed the absence of acute infection or ongoing inflammatory conditions. Plasma samples were collected at the time of diagnosis, before treatment, and before talc pleurodesis to eliminate potential confounding effects on the inflammatory response. Additionally, 17 surgically resected, formalin-fixed, and paraffin-embedded PM tissue specimens, collected at the Department of Thoracic Surgery, Medical University of Vienna, Austria, representing all histological subtypes, were used for in situ hybridization experiments. Demographic and clinical parameters of all patients were consecutively collected from hospital databases, including age, sex, tumor histology, disease stage, treatment strategies, and survival data. In all analyzed patients, PM diagnosis was histologically proven during routine clinical work-up. All interventions and treatment approaches were undertaken as part of routine clinical care.

This study was approved by the Ethics Committee of the Medical University of Vienna (EK 2137/2022) in accordance with the Declaration of Helsinki. Informed consent was obtained from all participants prior to the donation of biological materials.

### 2.2. Staging and Treatment

Patients were staged according to the eighth edition of the pleural mesothelioma tumor, node, metastasis (TNM) classification by the International Mesothelioma Interest Group (IMIG) and the International Association for the Study of Lung Cancer (IASLC) [35,36,37,38]. Computed tomography (CT) scans of the chest and abdomen, and brain magnetic resonance imaging (MRI) or cranial CT scans were used before 2015, and positron emission computed tomography scans (PET/CT) and brain MRI after 2015 for clinical staging. Stage I–II and stage III–IV were defined as early- and late-stage disease, respectively. Treatment strategies included multimodality treatment (MMT), systemic chemotherapy with or without palliative radiotherapy (CHT/RT), and best supportive care (BSC). Multimodality treatment consisted of neoadjuvant doublet chemotherapy with platinum and pemetrexed, subsequent surgery in terms of macroscopic complete resection with or without adjuvant hemithoracic radiation therapy. Patients not eligible for multimodality protocols were treated by systemic platinum-based chemotherapy combined with pemetrexed and received palliative radiotherapy in case of symptomatic metastases. BSC was defined as symptom relief, including pleural effusion and ascites management, as well as pain management and palliative care with no active cancer treatment to improve quality of life.

### 2.3. Determination of Circulating miR-21 Levels by qRT-PCR in Plasma

To investigate the presence of miR-21 in the blood, Exiqon’s miRCURY™ RNA Isolation Kit—Biofluids was used to isolate and purify RNA (Exiqon A/S, Vedbaek, Denmark) according to the manufacturer’s instructions. Isolated miRNAs were then quantified using the miRCURY LNA™ Universal RT microRNA PCR system (Exiqon A/S). Specifically, we used primers for miR-21 and miR-16, with the latter used as an endogenous reference for normalization [39]. Specifically, miR-21-5p and miR-16-5p were analyzed. Additionally, 0.5 µL of a synthetic spike-in (cel-miR-39), provided in the RNA isolation kit, was added to each plasma sample prior to RNA extraction and processed alongside miR-21 and miR-16. Samples were measured in triplets on an Applied Biosystems 7500 Fast Real-Time PCR System (Thermo Fisher Scientific, Waltham, MA, USA) as previously described [40]. Relative miR-21 expression levels were calculated by the ΔCt method as follows: ΔCt  =  mean value Ct (reference miR-16 or cel-miR-39) − mean value Ct of miRNA of interest (miR-21). Subsequently, ΔCt values were 2^−(ΔCt)^ transformed in order to obtain normal distribution data.

To minimize pre-analytical variability, plasma samples were processed according to standardized protocols across participating centers. Samples showing visible hemolysis were excluded. In addition, the endogenous reference miR-16 and the spike-in cel-miR-39 were used as quality controls to account for technical variability and potential hemolysis-related bias.

### 2.4. Detection of miR-21 in PM Tissues by In Situ Hybridization (ISH)

We performed in situ hybridization (ISH) according to the manufacturer’s protocol, as provided by Exiqon, and as previously described [41], with slight modifications. The provided sequence, 5′-TCAACATCAGTCTGATAAGCTA-3′, was a double-DIG labeled miRCURY LNA™ microRNA detection probe for miR-21-5p. The small nuclear RNA (snRNA) RNU6B (U6) was used as a positive control to detect nuclei, while a scrambled probe served as a negative control. All probes were purchased from Exiqon (Exiqon A/S).

Tissue sections (6 μm thick) were deparaffinized in ethanol solutions of 99%, 96%, and 70% and digested with Proteinase-K (15 μg/mL) for 10 min at 37 °C using an Abbott hybridizer System (Abbott Laboratories, Abbott Park, IL, USA). Then, LNA™ probes were denatured and diluted in Exiqon ISH buffer. Hybridization was performed with 40 nM miR-21 probe, 1 nM U6 probe, and 20 nM scrambled probe at 50 °C for 60 min, followed by stringent washes at 55 °C and incubation in blocking solution. The digoxigenins were recognized by a specific anti-DIG antibody diluted in blocking solution containing sheep serum and directly conjugated with the enzyme Alkaline Phosphatase (AP). After PBS washing, samples were stained with a freshly prepared NBT/BCIP substrate reagent containing 0.2 mM Levamisole (2 h at 30 °C), and the slides were incubated in KTBT buffer twice to obtain a blue staining. Nuclear Fast Red (Vector Laboratories, Newark, CA, USA) was used as a counterstain for 1 min. Sections were analyzed microscopically, and representative images were taken using a Nikon Eclipse 80i microscope and DS-Fi1 camera (Nikon, Tokyo, Japan).

### 2.5. Measurement of SMRP in Serum

Soluble mesothelin-related protein (SMRP) was quantified using the Lumipulse G1200 System (Fujirebio Europe, Ghent, Belgium), which is a fully automated chemiluminescent enzyme immunoassay. Experiments were performed according to the manufacturer’s instructions.

### 2.6. Statistical Analysis

Categorical data were displayed as counts and percentages, and metric data as median and interquartile range (IQR), or, in the case of survival, as median and corresponding 95% confidence interval (CI) if not otherwise indicated. For all statistical analyses of miR-21 levels, transformed ΔCt (miR-16 − miR-21) was used. Patients were dichotomized into high and low pre-treatment miR-21 relative expression level groups by the median normalized value (0.401) and by the median SMRP concentration (1.80 nmol/L), as commonly applied in exploratory biomarker studies lacking established biological cut-off values. To compare groups, the Mann–Whitney U test, the Kruskal–Wallis test, or the Chi-square test was used as appropriate. The correlation of metric data was analyzed by Pearson’s correlation coefficient. Overall survival was defined as the time from diagnosis to death or to the last follow-up. Survival was estimated by the Kaplan–Meier method and log-rank test. Univariable and multivariable Cox regression analyses were used to evaluate the effects of potential influencing factors, including age, sex, histology, stage, treatment, SMRP, and circulating miR-21 levels. In the full cohort analyzed for circulating miR-21 (*n* = 94), a total of 77 events occurred during follow-up. Considering six clinically relevant covariates (age, sex, histology, disease stage, treatment, and circulating miR-21), this corresponds to approximately 12.8 events per variable, indicating adequate model stability for exploratory prognostic analysis. In the subgroup of patients with available SMRP measurements included in the combined multivariable model (*n* = 84), 69 events were observed. Including seven covariates (age, sex, histology, disease stage, treatment, SMRP, and circulating miR-21) resulted in approximately 9.9 events per variable, which is considered acceptable for multivariable survival analysis in rare diseases. Differences were considered statistically significant for *p*-values < 0.05. Statistical analyses were performed using SPSS 30.0 software (SPSS Inc., Chicago, IL, USA), and plots were generated using GraphPad Prism version 8.4.3. (GraphPad Software, Boston, MA, USA).

## 3. Results

### 3.1. Patient Characteristics

In this current analysis, we investigated circulating plasma miR-21 in relation to clinical patient characteristics, serum SMRP levels, and survival to assess its potential suitability as a biomarker in PM.

In total, for miR-21 quantification, plasma samples from 94 patients (74 male, 79%) from three different institutions with histologically verified pleural mesothelioma were included. The median age was 64 years (IQR: 56–64). Epithelioid histology made up 80% (75/94) of all cases. Thirty-seven patients (39%, 37/94) were diagnosed in early stages (Stage I and II, 8th TNM classification), and 57 patients (61%, 57/94) presented in advanced stages (Stage III and IV, 8th TNM classification). Treatment approaches included multimodality treatment (51%, 48/94), chemotherapy with or without palliative radiotherapy (39%, 37/94), and best supportive care (10%, 9/94). For SMRP measurement, serum samples from 84 patients (66 male, 79%) of the same cohort were available. Baseline characteristics of all PM patients are displayed in Table 1 and Table 2.

### 3.2. Circulating miR-21 Is Elevated in Patients with Pleural Mesothelioma

**Figure 1:**
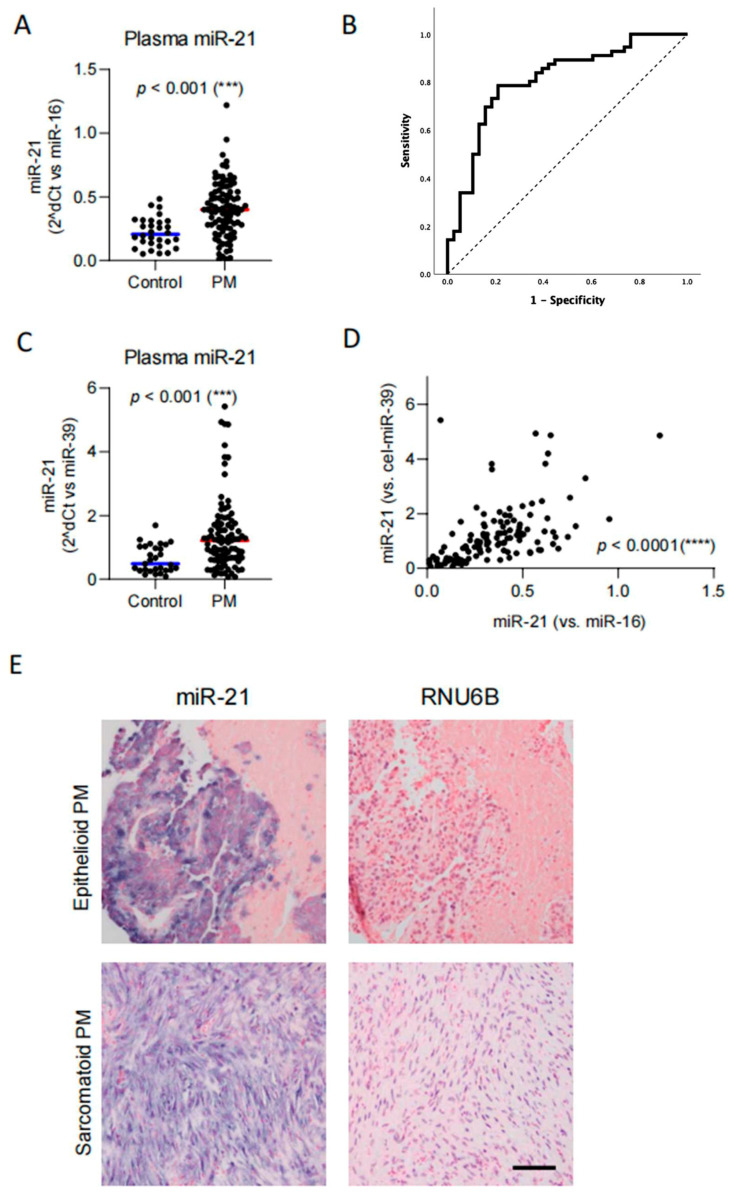
MiR-21 is elevated in PM. (**A**) Normalized circulating miR-21 levels (2^−(ΔCt)^, miR-16-miR-21) in PM patients and healthy controls. Each dot represents one patient, and medians are shown as horizontal lines. Mann–Whitney U test, *** *p* < 0.001. (**B**) ROC-curve demonstrating discrimination between PM patients and healthy controls with an AUC of 0.809 (95% CI: 0.718–0.900, *p* < 0.001), corresponding to a sensitivity of 74% (95% CI: 64.3–83.3) and a specificity of 76% (95% CI: 58.8–88.2). (**C**) Normalized circulating miR-21 levels (2^−(ΔCt)^, cel-miR-39-miR-21) in PM patients and healthy controls. Each dot represents one patient, and medians are shown as horizontal lines. Mann–Whitney U test, *** *p* < 0.001. (**D**) Correlation between miR-21 levels, normalized to either miR-16 or cel-miR-39. Pearson r: 0.53, **** *p* < 0.0001. (**E**) Representative images of in situ hybridization (ISH) showing miR-21 expression and the nuclear RNU6B (blue) in epithelioid and sarcomatoid PM. Nuclear Fast Red (pink) was used as a counterstain. Scale bar: 100 µm.

Circulating miR-21 levels (relative to plasma miR-16) were compared between PM patients and healthy individuals and found to be significantly elevated in PM patients (*n* = 94, median normalized value 0.40, IQR: 0.26–0.54) when compared to healthy controls (*n* = 30; median normalized value 0.21, IQR: 0.14–0.31; *p* < 0.001) (Figure 1A). For both miR-21 and miR-16, the 5p strand is considered the predominant and functionally active guide strand; the majority of published qPCR biomarker studies have standardized on detecting miR-21-5p, facilitating comparability across studies [42,43,44,45,46]. Accordingly, in our study, we also used probes for miR-21-5p and miR-16-5p, referred to as miR-21 and miR-16, respectively.

Receiver operating characteristic (ROC) curve analysis was performed to assess the ability of miR-21 to discriminate between patients with PM and healthy individuals, and showed an area under the curve (AUC) of 0.809, indicating that plasma miR-21 has a sensitivity of 74% (95% CI: 64.3–83.3) and a specificity of 76% (95% CI: 58.8–88.2; *p* < 0.001) to identify PM patients (Figure 1B). Of note, when miR-21 was normalized to the spiked-in cel-miR-39, similar results were obtained, showing significantly higher miR-21 levels in PM patients (*n* = 94, median normalized value 1.27, IQR: 0.63–1.75) compared to controls (*n* = 30; median normalized value 0.48, IQR: 0.28–1.02; *p* < 0.001) (Figure 1C). Also, we found a significant correlation between the two transformed ΔCt values (Pearson r: 0.53, *p* < 0.0001) (Figure 1D). In all further analyses, plasma miR-21 levels are shown as transformed ΔCt (miR-16 − miR-21).

### 3.3. MiR-21 Is Present in Tumor Cells

Next, to verify that circulating miR-21 indeed originates from tumor cells, we performed in situ hybridization in matched PM tissue specimens. We detected a strong miR-21 signal (blue) in tumor cells, as shown in representative pictures of epithelioid and sarcomatoid PM (Figure 1E). However, due to the limited sample size, no significant correlation between tissue and plasma miR-21 levels was observed.

### 3.4. Circulating miR-21 Levels Do Not Correlate with Histology, Disease Stage, Sex, Age, and SMRP

**Figure 2:**
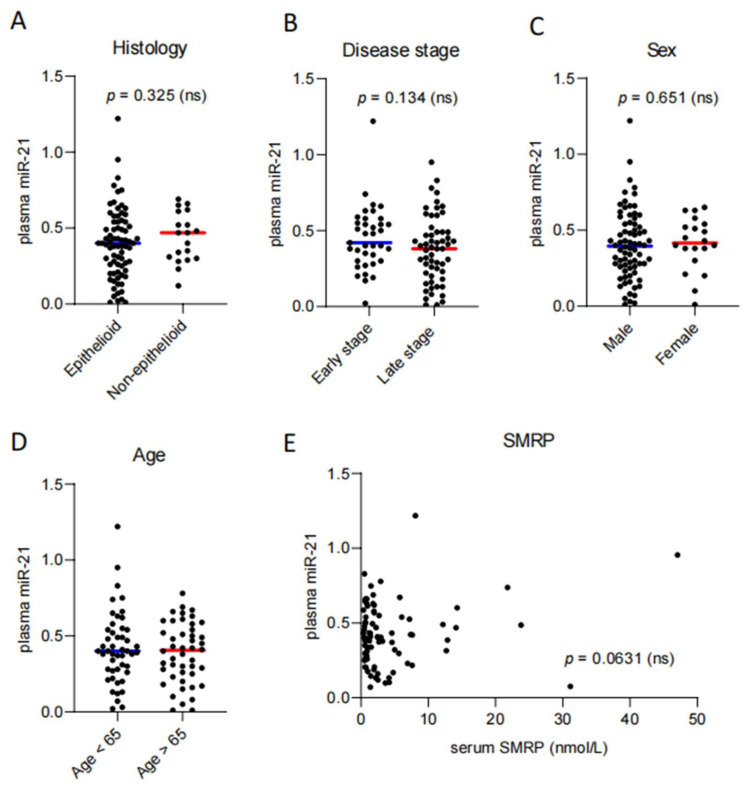
MiR-21 is not associated with histology, disease stage, sex, age, and SMRP. Comparison of normalized circulating miR-21 levels (2^−(ΔCt)^, miR-16-miR-21) between (**A**) patients with epithelioid and non-epithelioid mesothelioma (*p* = 0.325), (**B**) early- and late-stage disease (*p* = 0.134), (**C**) male and female patients (*p* = 0.651), and (**D**) older and younger age groups (*p* = 0.751). Each dot represents one patient, and medians are shown as horizontal lines. Mann–Whitney U test. ns: not significant. (**E**) Correlation between plasma miR-21 and serum SMRP levels. Pearson r: 0.20, *p* = 0.0631. ns: not significant.

The expression of circulating miR-21 levels between the epithelioid group (75/94, median normalized value 0.40, IQR: 0.22–0.54) and the non-epithelioid group (19/94, median normalized value 0.47, IQR: 0.30–0.61) did not show significant differences (*p* = 0.325) (Figure 2A). Also, no significant differences in circulating miR-21 levels were observed between early-stage (37/94, median normalized value 0.42, IQR: 0.30–0.55) and late-stage disease (57/94, median normalized value 0.38, IQR: 0.22–0.49; *p* = 0.134) (Figure 2B). Furthermore, no significant differences were found between male (74/94, median normalized value 0.39, IQR: 0.26–0.54) and female (20/94, median normalized value 0.42, IQR: 0.34–0.53; *p* = 0.651) (Figure 2C) patients, as well as between older (46/94, ≥65 years, median normalized value 0.41, IQR: 0.25–0.54) and younger patients (48/94, <65 years, median normalized value 0.40, IQR: 0.27–0.54; *p* = 0.751) (Figure 2D).

Additionally, to test miR-21 against soluble mesothelin-related protein (SMRP), which is the only accepted and established prognostic marker for PM, we measured serum SMRP in 84 patients (median value 1.80 nmol/L, IQR: 0.77–4.61) from our cohort. Interestingly, we found no significant correlation between the two markers (Pearson r: 0.21, *p* = 0.0631) (Figure 2E), indicating an independent role of miR-21.

### 3.5. Circulating miR-21 Is an Independent Prognostic Factor in Pleural Mesothelioma

As a next step, we tested whether levels of circulating miR-21 correlate with patient prognosis. Patients were divided into high and low circulating miR-21 expression level groups by the median normalized value (0.401). Between the high and low miR-21 groups, no significant differences in age (*p* = 0.856), sex (*p* = 0.614), histology (*p* = 0.441), stage (*p* = 0.527), and treatment (*p* = 0.265) were observed (Table 1).

**Figure 3:**
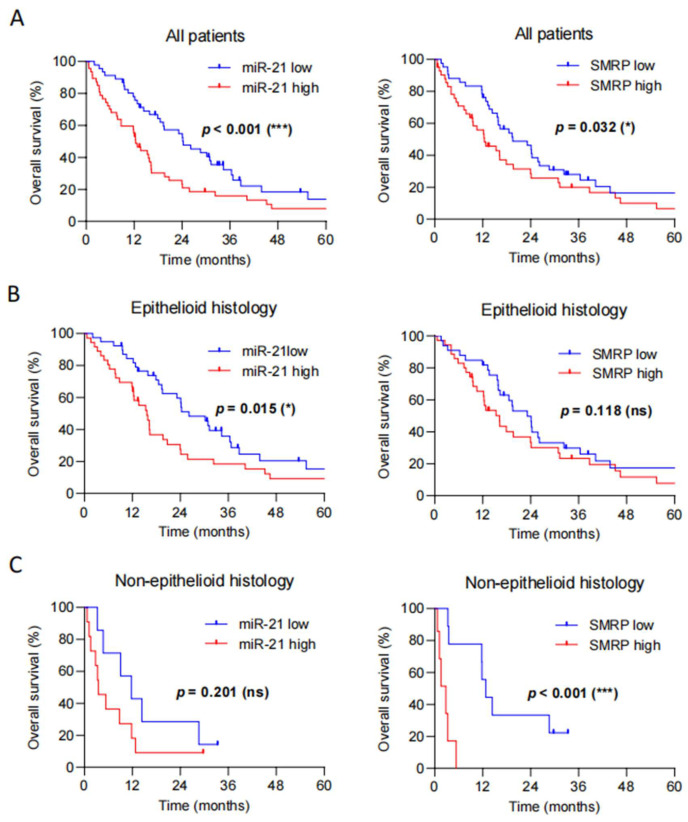
High-circulating miR-21 levels are associated with significantly shorter median overall survival in pleural mesothelioma. Patients were dichotomized into low (blue) and high (red) miR-21 and SMRP groups by the median normalized levels of circulating miR-21 (0.401) and the median concentration of SMRP (1.80 nmol/L), respectively. Kaplan–Meier analyses were performed for miR-21 (*n* = 94) and SMRP (*n* = 84). (**A**) OS was shorter in patients with high miR-21 levels (24.3 vs. 12.4 months; HR 1.79, 95% CI: 1.14–2.81; *p* < 0.001). For SMRP, OS was 19.5 vs. 12.4 months (HR 1.55, 95% CI: 0.96–2.49; *p* = 0.032). (**B**) In epithelioid histology, OS was 26.2 vs. 15.6 months for low versus high miR-21 (HR 1.71, 95% CI: 1.03–2.84; *p* = 0.015). (**C**) In non-epithelioid histology, OS was 11.8 vs. 3.5 months for low versus high miR-21 (HR 1.94, 95% CI: 0.69–5.43; *p* = 0.201). Low SMRP was associated with longer OS (12.8 vs. 2.8 months; HR 12.48, 95% CI: 2.36–65.91; *p* < 0.001). Low SMRP was associated with a significantly prolonged OS in non-epithelioid disease (12.8 vs. 2.8 months, HR 12.48, 95% CI: 2.36–65.91, *p* < 0.001). Statistical significance was defined as follows: * *p* < 0.05; *** *p* < 0.001; ns, not significant.

Median OS for the entire cohort was 16.3 months (*n* = 94, 95% CI: 12.29–20.24). Patients with low miR-21 levels had a significantly longer overall survival when compared to those with high miR-21 levels (24.3 vs. 12.4 months, HR 1.79, 95% CI: 1.14–2.81, *p* < 0.001) (Figure 3A left). In epithelioid disease, high miR-21 levels were associated with a significantly worse prognosis (OS 15.6 vs. 26.2 months in patients with low vs. high miR-21 levels, respectively; HR 1.71, 95% CI: 1.03–2.84, *p* = 0.015) (Figure 3B left). However, among the small number of patients with non-epithelioid PM, no significant survival differences were observed between patients with low and high miR-21 levels (OS 11.8 vs. 3.5 months; HR 1.94, 95% CI: 0.69–5.43, *p* = 0.201) (Figure 3C left).

Regarding SMRP levels, patients were dichotomized by the median serum SMRP value of 1.80 nmol/L. A significant difference between the high and low SMRP groups was observed in disease stage (*p* = 0.046), but not in age (*p* = 0.513), sex (*p* = 0.917), histology (*p* = 0.578), or treatment (*p* = 0.194) (Table 2). Specifically, patients with high SMRP levels more frequently presented with late-stage disease, consistent with the established association of SMRP with tumor burden. Median OS for this cohort was 16.2 months (*n* = 84, 95% CI: 12.82–23.19). As expected, high SMRP was connected to worse prognosis in all patients (OS 19.5 vs. 12.4 months; HR 1.55, 95% CI: 0.96–2.49, *p* = 0.032) (Figure 3A, right). In contrast to miR-21, high SMRP levels showed no difference in epithelioid disease (OS 23.2 vs. 16.2 months; HR 1.43, 95% CI: 0.85–2.42., *p* = 0.118) (Figure 3B, right), but were associated with significantly worse overall survival in patients with non-epithelioid disease (OS 12.8 vs. 2.8 months; HR 12.48, 95% CI: 2.36–65.91, *p* < 0.001) (Figure 3C, right).

Finally, potential prognostic factors were assessed using univariable analysis, including age, sex, histological subtypes, circulating miR-21 levels, SMRP levels, disease stage, and type of treatment. Subsequently, multivariable analysis was performed to identify independent predictors for survival. In the univariable analysis, the following parameters were predictors for OS: epithelioid histology (HR 2.25, 95% CI: 1.21–4.19, *p* = 0.008), miR-21 levels (HR 1.64, 95% CI: 1.10–2.64, *p* = 0.041), SMRP levels (HR 1.55, 95% CI: 0.96–2.49, *p* = 0.032), disease stage (HR 2.94, 95% CI: 1.77–4.89, *p* < 0.001) and treatment (HR 1.96, 95% CI: 1.17–3.29, *p* < 0.001). Importantly, in the multivariable analysis, only miR-21 (HR 3.12, 95% CI: 1.78–5.47, *p* < 0.001), but not SMRP (HR 1.58, 95% CI: 0.93–2.66, *p* = 0.090) was confirmed as an independent predictor for survival, alongside histology (HR 2.09, 95% CI: 1.11–3.95, *p* = 0.024) and treatment (HR 2.37, 95% CI: 1.18–4.78, *p* < 0.001) (Table 3).

## 4. Discussion

Due to the late onset and rapid progression of pleural mesothelioma, which often results in diagnosis in advanced stages with a dismal prognosis, several studies have concentrated on the search for non-invasive diagnostic markers with the potential for earlier diagnosis and screening in high-risk populations with a previous history of asbestos exposure [47,48]. Our findings demonstrate that miR-21 may serve as a potential diagnostic and prognostic, non-invasive, and reproducible biomarker for pleural mesothelioma.

MiR-21 was among the first of many miRNAs identified as an oncogenic miRNA. Since then, it has emerged as a promising biomarker in various malignancies due to its role in cancer development, tumor growth, progression, prognosis, and even treatment prediction [28,49]. In cancer tissue, the upregulation of miR-21 has been shown to correlate with poorer survival in pancreatic, breast, gastric, and kidney cancer [49,50,51,52]. In pleural mesothelioma tissue, the first systemic investigation of microRNA expression by Kirschner et al. identified a signature score based on six microRNAs, including miR-21-5p, miR-23a-3p, miR-30e-5p, miR-221-3p, miR-222-3p, and miR-31-5p, which predicted survival with high accuracy in patients undergoing cytoreductive surgery. This novel miR-score showed added value in combination with well-established clinical prognostic factors, such as histology, sex, and age, to accurately classify patients likely to benefit from radical multimodality treatment protocols [53].

The overexpression of miR-21 in pleural mesothelioma has been demonstrated in cell lines and tissue samples. It has been shown to exhibit an oncogenic function by targeting tumor suppressor genes, including programmed cell death protein 4 (PDCD4) [24,54]. The association between treatment, survival, and miR-21 expression in tissue specimens, however, remains a subject of investigation. Kirschner et al. found that a novel miR-Score, using expression data of 6 microRNAs, including miR-21, in tissue specimens after extrapleural pneumonectomy in 16 patients, predicted prognosis. Higher miR-21 expression in tissue was also independently associated with shorter overall survival [53].

The role of miR-21 in blood and pleural effusion of pleural mesothelioma patients has been particularly underreported and has only been investigated in small cohorts. In pleural effusion, an overexpression of miR-21 was observed in 29 cytology samples [55]. Similarly, levels of miR-21 expression in blood and pleural effusion were found to be upregulated in 20 patients with pleural mesothelioma [56]. The expression of miR-21 and its correlation with prognosis have only been described in other malignancies [57,58,59].

While the use of circulating miR-21 as a potential non-invasive biomarker has also been investigated across different cancer types for diagnosis and disease monitoring, its clinical role requires further validation due to inconsistencies in accuracy and specificity [29,60,61]. The upregulation of circulating miR-21 has been reported in a wide range of malignancies, particularly breast, colorectal, gastric, pancreatic, prostate, hepatocellular, and non-small cell lung cancer [43,49,62,63,64,65,66,67]. Circulating miR-21 was found to be overexpressed in breast cancer patients and associated with poor outcomes [68,69,70]. In lung cancer, circulating miR-21 was strongly associated with increased metastatic risk, particularly with the development of bone metastases. Additionally, higher expression was correlated with poorer survival, higher disease stage, and an increased risk of lymph node metastases [71,72,73,74,75,76]. MiR-21 in the blood of patients with colorectal carcinoma has been reported to be consistently overexpressed with a moderate to high diagnostic and prognostic value [77,78,79,80,81]. Evidence also suggests the diagnostic significance of circulating miR-21 in pancreatic adenocarcinoma [43,82,83]. Despite its broad overexpression in several malignancies, circulating miR-21 lacks disease specificity. Research indicates that plasma miR-21 may serve as a non-specific, non-invasive diagnostic biomarker in early stages of lung, colorectal, and breast cancer [67].

The diagnosis of pleural mesothelioma frequently poses significant clinical challenges due to the need for invasive procedures to obtain adequate tissue biopsies required for definitive diagnosis. While various biomarkers have been investigated, none have been established as a non-invasive diagnostic biomarker in routine clinical practice. The most studied circulating protein markers for PM are mesothelin/SMRP (soluble mesothelin-related peptides), calretinine, osteopontin, fibulin-3, HMGB1 (High Mobility Group Box 1), and VEGF (vascular endothelial growth factor) [6,10,84,85]. Our group and others have previously reported that circulating activin A is elevated in pleural mesothelioma, associated with a reduced response to platinum-based chemotherapy [86], and may be used as a potential biomarker for differentiating PM from other thoracic diseases, as well as supporting histological classification with a prognostic value in epithelioid subtypes [87]. We have also identified circulating fibrinogen as an independent prognostic and predictive marker in PM [88]. Furthermore, we have investigated the clinical relevance of circulating complement component 4d (C4d) as well and found that high C4d levels were associated with higher tumor volume, treatment resistance, and shorter overall survival [89]. Nevertheless, SMRP remains the most extensively studied circulating biomarker in pleural mesothelioma and is widely used in clinical practice for disease monitoring. However, SMRP primarily reflects tumor burden and mesothelin expression and has shown limited independent prognostic value across heterogeneous patient populations [13,14,15]. In the present study, although elevated SMRP levels were associated with shorter survival in univariable analysis, SMRP failed to retain independent prognostic significance in the multivariable analysis.

While several circulating miRNAs are dysregulated in pleural mesothelioma, including the upregulation of miR-197-3p, miR-1281, miR-32-3p, miR-101, miR-25, miR-26b, miR-335, and miR-433, as well as the downregulation of miR-126, miR-191, and miR-223, they showed moderate accuracy with ongoing research to validate these biomarkers for the diagnosis and management of pleural mesothelioma. The primary focus of miR-21 research has often been on tissues, with scant attention to cytological samples, while direct investigation of miR-21 levels in blood for pleural mesothelioma patients is limited. Moreover, the correlation between circulating miR-21 and outcome has not been markedly investigated to date. Herein, we demonstrated that high miR-21 levels were associated with significantly shorter overall survival (12.4 vs. 24.3 months, *p* < 0.001). Additionally, high-circulating miR-21 was confirmed as an independent predictor of poor survival in multivariable analysis that accounts for established clinical covariates (*p* < 0.001). Our findings are uniquely strengthened by validation through in situ hybridization, which directly confirms miR-21 expression within tumor tissues and links circulating levels to their biological source.

Overall, circulating miR-21 emerged as a robust and independent prognostic biomarker, even when directly compared with SMRP within the same multivariable model of a relatively large cohort of 84 matched patients. The lack of correlation between miR-21 and SMRP suggests that miR-21 captures tumor-biological and host-response mechanisms beyond mesothelin shedding alone, potentially reflecting oncogenic signaling and tumor-immune interactions. While earlier studies have primarily focused on tissue-based microRNA signatures or small cytological cohorts, evidence linking circulating miR-21 levels to clinical outcome in pleural mesothelioma has been sparse and limited by a lack of biological source confirmation. Integration of miR-21 into the existing biomarker framework of pleural mesothelioma suggests that miR-21 represents a distinct biological layer compared with previously investigated circulating proteins. Whereas activin A and fibrinogen predominantly capture systemic and microenvironment-driven processes, miR-21, as a post-transcriptional regulator, may more directly capture tumor-intrinsic regulatory mechanisms associated with oncogenic signaling. The absence of correlation between miR-21 and SMRP further supports the concept that miR-21 provides non-redundant biological information. This distinction is clinically relevant, as it raises the possibility that circulating miRNAs and protein-based markers interrogate complementary aspects of mesothelioma biology. Consequently, combined biomarker strategies incorporating miR-21 alongside circulating proteins may provide more comprehensive disease characterization than single-marker approaches.

The study’s limitations include its retrospective design, the lack of an external validation cohort, the small number of subjects with non-epithelioid histology and healthy controls, and the absence of clinically relevant disease-control cohorts. Also, the number of tissue samples available for in situ hybridization was limited and did not allow quantitative correlation analysis between tissue and circulating miR-21 levels. While the overall sample size is comparatively large for a biomarker study in this rare disease, these findings should be interpreted with caution. In particular, miR-21 is not disease-specific and therefore should not be interpreted as a stand-alone diagnostic biomarker for pleural mesothelioma. Its potential clinical value may lie primarily in its prognostic signal and in its integration with established circulating markers, such as SMRP, or with relevant clinical parameters within multimarker strategies. Prospective validation in independent cohorts, including clinically relevant differential diagnostic populations, will be required to confirm clinical applicability. As miR-21 is broadly dysregulated across multiple malignancies and inflammatory conditions, disease specificity remains limited, reinforcing the need for multimarker strategies integrating molecular and clinical parameters.

Overall, in this multicenter study comprising a comparatively large cohort of 94 patients with pleural mesothelioma, we identified circulating miR-21 as a potential biomarker capable of reliably distinguishing patients from healthy controls, with an AUC of 0.81. A key strength and distinguishing feature of our study is the validation of miR-21 expression directly within tumor cells by in situ hybridization, thereby providing biological confirmation that circulating miR-21 reflects tumor-derived signals rather than nonspecific systemic inflammation. Together, these findings position circulating miR-21 as a biologically supported liquid biopsy biomarker with independent prognostic value and a measurable diagnostic signal in pleural mesothelioma. Importantly, when evaluated for prognostic utility, miR-21 emerged as one of the strongest independent predictors of overall survival with a hazard ratio of 3.12 on multivariable analysis, outperforming SMRP. Therefore, these findings highlight the potential of miR-21 as a valuable and clinically relevant circulating biomarker, integrating diagnostic utility, independent prognostic power, and tumor specificity in pleural mesothelioma.

## Figures and Tables

**Table 1:** Clinicopathological characteristics of PM patients grouped by circulating miR-21.

Demographics	Study Cohort (*n* = 94)	Low miR-21 (*n* = 47)	High miR-21 (*n* = 47)	*p*-Value
Age, years, median, IQR	64 (56–64)	64 (55–71)	65 (58–71)	0.856
Sex				0.614
Male	74 (79%)	38 (81%)	36 (77%)	
Female	20 (21%)	9 (19%)	11 (23%)	
Histology				0.441
Epithelioid	75 (80%)	39 (83%)	36 (77%)	
Non-epithelioid	19 (20%)	8 (17%)	11 (23%)	
Stage				0.527
Early	37 (39%)	17 (36%)	20 (43%)	
Late	57 (61%)	30 (64%)	27 (57%)	
Treatment				0.265
MMT	48 (51%)	22 (47%)	26 (55%)	
CHT +/− RTH	37 (39%)	22 (47%)	15 (32%)	
BSC	9 (10%)	3 (6%)	6 (13%)	

Abbreviations: PM, pleural mesothelioma; miR-21, microRNA-21; IQR, interquartile range; MMT, multimodality treatment; CHT, chemotherapy; RTH, radiotherapy; BSC, best supportive care.

**Table 2:** Clinicopathological characteristics of PM patients grouped by circulating SMRP.

Demographics	Study Cohort (*n* = 84)	Low SMRP (*n* = 42)	High SMRP (*n* = 42)	*p*-Value
Age, years, median, IQR	64 (55–71)	63 (54–69)	66 (57–75)	0.513
Sex				0.917
Male	66 (79%)	33 (79%)	33 (79%)	
Female	18 (21%)	9 (21%)	9 (21%)	
Histology				0.578
Epithelioid	68 (81%)	33 (79%)	35 (83%)	
Non-epithelioid	16 (19%)	9 (21%)	7 (17%)	
Stage				0.046
Early	35 (42%)	22 (52%)	13 (31%)	
Late	49 (58%)	20 (48%)	29 (69%)	
Treatment				0.194
MMT	42 (50%)	25 (60%)	17 (41%)	
CHT +/− RTH	33 (39%)	14 (33%)	19 (45%)	
BSC	9 (11%)	3 (7%)	6 (14%)	

Abbreviations: PM, pleural mesothelioma; SMRP, soluble mesothelin-related protein; IQR, interquartile range; MMT, multimodality treatment; CHT, chemotherapy; RTH, radiotherapy; BSC, best supportive care.

**Table 3:** Univariable and multivariable survival subgroup analyses of pleural mesothelioma patients with available miR-21 and SMRP levels.

Variables			Univariable			Multivariable		
	*n* = 84	OS (CI)	*p*-Value	HR	95% CI	*p*-Value	HR	95% CI
Age			0.487	1.18	0.74–1.90	0.512	1.01	0.59–1.71
>65	41	15.6 (11.1–20.1)						
<65	43	18.5 (14.1–22.9)						
Sex			0.357	1.33	0.72–2.44	0.935	1.01	0.53–1.92
Male	66	17.9 (13.9–21.9)						
Female	18	16.2 (10.8–21.6)						
Histology			0.008	2.25	1.21–4.19	0.024	2.09	1.11–3.95
Epithelioid	68	18.5 (14.9–22.2)						
Non-epithelioid	16	5.4 (0.0–16.0)						
MiR-21 levels			0.041	1.64	1.10–2.64	<0.001	3.12	1.78–5.47
High	42	12.4 (10.2–14.6)						
Low	42	24.3 (18.3–29.9)						
SMRP levels			0.032	1.55	0.96–2.49	0.090	1.58	0.93–2.66
High	42	12.4 (6.7–18.1)						
Low	42	19.5 (10.2–28.8)						
Stage			<0.001	2.94	1.77–4.89	0.068	2.79	1.25–6.26
Early	35	24.2 (22.1–26.3)						
Late	49	11.8 (8.1–15.6)						
Treatment			<0.001	1.96	1.17–3.29	<0.001	2.37	1.18–4.78
MMT	42	24.0 (18.4–30.0)						
CTH +/− RTH	33	13.2 (9.2–17.2)						
BSC	9	2.8 (2.0–3.5)						

Abbreviations: OS, overall survival; CI, confidence interval; HR, hazard ratio; MMT, multimodality treatment; CTH, chemotherapy; RTH, radiotherapy; BSC, best supportive care; miR-21, microRNA-21; SMRP, soluble mesothelin-related protein.

## Data Availability

The data that support the findings of this study are available on request from the corresponding author (K.Sc.) upon reasonable request. The data are not publicly available due to privacy of the research participants.
